# Challenges Faced by Cancer Patients in Receiving Health Services During the COVID-19 Pandemic in a Tertiary Care Hospital in Rajasthan

**DOI:** 10.7759/cureus.34491

**Published:** 2023-02-01

**Authors:** Prasannajeet Bal, Neha Mantri, Akhil D Goel, Nitin K Joshi, Yogesh K Jain, Puneet Pareek, Manoj K Gupta, Bharti Devnani, Akanksha Solanki, Pankaj Bhardwaj

**Affiliations:** 1 School of Public Health, All India Institute of Medical Sciences, Jodhpur, Jodhpur, IND; 2 Community Medicine and Family Medicine, All India Institute of Medical Sciences, Jodhpur, Jodhpur, IND; 3 Radiation Oncology, All India Institute of Medical Sciences, Jodhpur, Jodhpur, IND

**Keywords:** health care facilities, preventive health, oncology, challenges, vaccination, cancer, covid-19

## Abstract

Background: Amidst the COVID-19 pandemic, cancer patients may have faced difficulty accessing health care. This study explored the challenges experienced by cancer patients in availing of healthcare during the pandemic, as well as the vaccination status and prevalence of COVID-19 infection among cancer patients in the year 2021.

Method: A cross-sectional study was conducted in a tertiary care hospital in Jodhpur, Rajasthan, to interview 150 patients from the oncology department using convenience sampling. Face-to-face interviews lasted for 20-30 minutes. The first segment of the pretested semi-structured questionnaire was directed at obtaining the patient's socio-demographic characteristics, while the second segment focused on the problems that patients encountered during the pandemic in receiving cancer care. The data were analyzed using Statistical Packages for Social Sciences (SPSS) software (IBM Corp., Armonk, NY).

Results: Several constraints, such as a lack of transportation services, difficulty in availing outpatient department (OPD) and teleconsultation services, long waiting times, and deferred surgeries and therapies, have hampered cancer care. COVID-19 mitigation measures further imposed additional stress and financial burden on cancer patients. Moreover, there was low vaccination coverage among cancer patients, which increases their probability of acquiring an infection.

Conclusion: Policy reforms must prioritize cancer care in India to maintain a continuum of care by ensuring medication, teleconsultation, uninterrupted treatment, and complete vaccination to decrease the risk of COVID-19 infection and facilitate patient compliance with the healthcare delivery system.

## Introduction

Since the end of 2019, the entire globe has been facing an unprecedented health crisis that has led to the diversion of maximum resources to COVID-19 management. Cancer is a broad group of diseases characterized by abnormal and uncontrolled cell development and it is also the world's second major cause of death, accounting for an estimated 9.6 million deaths yearly [1]. On the other hand, COVID-19, an infectious disease caused by a coronavirus that originated in Wuhan, China, causes a pneumonia-like illness with a serious impact on the elderly and individuals with comorbidities like heart disease, respiratory disease, diabetes, and cancer [2].

Cancer treatment requires a multimodal approach, starting with early diagnosis and prompt treatment with multiple visits for therapies. The cancer community is having a difficult time acknowledging the paradoxical need to keep away from healthcare facilities amid pandemics. Ultimately, the hampered healthcare delivery kept the cancer community at an increased risk of morbidity, poor survival, and even death.

Because the whole world relied on the advent of a safe and effective vaccine to get rid of this deadly virus, it became essential for individuals who are much more susceptible to COVID-19-related illness, such as healthcare professionals, the elderly, and immunocompromised individuals with pre-existing medical conditions, to receive the primary care along with the timely vaccination during the pandemic [3].

Previous research has found that, when compared to the general population, cancer patients are three times more likely to suffer from COVID-19 because cancer and its treatments can weaken their immune systems [4]. There are limited studies that have discussed the impact of COVID-19 on the cancer care delivery system in the Indian context.

The purpose of this study was to identify the challenges that cancer patients faced during the COVID-19 pandemic in receiving healthcare services. In addition, the percentage of COVID-19 vaccination status among cancer patients was determined. We also calculated the post-vaccination positivity rate in cancer patients. In addition, we investigated the prevalence of influenza-like illness (ILI) among cancer patients for the year 2021.

## Materials and methods

A cross-sectional study was done at a tertiary care hospital in Jodhpur (Rajasthan) among the patients approaching the oncology department who had been diagnosed with cancer. The study was carried out after the Institutional Ethics Committee approved the study protocol (Certificate Reference Number: AIIMS/IEC/2021/3504). The confidentiality of patient information and the anonymity of data were maintained. The reporting of the study findings is done in line with the Strengthening the Reporting of Observational Studies in Epidemiology (STROBE) checklist. A total of 150 patients were enrolled from June 1 to July 31, 2021 (time-bound) based on convenience sampling from the patient register. Those patients who gave written consent for participation were included in the study. A face-to-face interview was conducted for 20-30 minutes. The first segment of the pretested semi-structured questionnaire was directed at obtaining the patient's socio-demographic characteristics and inquiries about the medical conditions, while the second segment focused on the problems that patients encountered during the pandemic in receiving cancer care. Every patient was asked about their vaccination status and infection with COVID-19 in the previous year. Further, patients were assessed about their mental or psychological status (depression) by the Patients' Health Questionnaire-2 (PHQ-2). The Patients' Health Questionnaire-2 inquires about the frequency of depressive moods over the prior two weeks [5]. Microsoft Excel (Microsoft® Corp., Redmond, WA) was used to enter the data and analyze it using Statistical Packages for Social Sciences (SPSS) version 23 software (IBM Corp., Armonk, NY) [6]. The mean, standard deviation, and percentage were used to summarize quantitative data for the study outcomes.

## Results

A total of 150 patients were interviewed for the study. Their mean age was 51.98 years, with a standard deviation of 14.1 years. Seventy-one (47.3%) of the study participants were females.

Challenges faced in availing transportation during the pandemic time

As the pandemic is almost new to everyone in society, everyone might have faced some problems and challenges at some point in time. The challenges range from transportation problems to the availability of care. At least more than half of the sampled population faced transportation problems from their residence to the hospital during the period of lockdown. Only a few of the patients have a personal vehicle (37.3%), whereas more than 60% rely on public transport, which is unavailable during the lockdown. Around 62.7% (n=94) of patients in our study faced transportation challenges. Moreover, 110 patients (73.3%) faced financial hardship to avail transportation to higher centres for cancer care.

Challenges faced by patients in seeking consultation

Because of the high infectivity of COVID-19, only a limited number of attendants are allowed inside the hospital with patients, so around half of the patients' attendants and caregivers faced restrictions during their hospital visits and stays. Table 1 represents the challenges faced by cancer patients in seeking consultations and treatment. Almost half of the sampled cancer patients who approached the oncology department faced long waiting times (>60-90 minutes). During the lockdown, the physical outpatient department (OPD) was replaced by teleconsultation. Almost 3/4th of the sampled population had difficulty getting a slot for teleconsultation due to network issues, long waiting times over the phone, and a heavy load in the telemedicine department. When the peak of the second wave started declining, most of the patients started approaching OPD. During this time, due to limited OPD patient intake, around half of the sampled population faced trouble getting access to OPD facilities. Around 25.3% of the sampled patients were not able to avail themselves of chemotherapy in daycare, whereas 6% of the cancer patients could not avail themselves of radiotherapy in daycare. Approximately 14.7% of the patients experienced deferment in surgery. More than 3/4th of the patients continued with the same treatment facility during the lockdown. However, less than a quarter of the sampled population was diverted towards private healthcare facilities either for some advanced care or due to time constrain.

**Table 1:** Challenges faced by patients in seeking consultation and treatment

	Frequency % (n = 150)
Restriction on visitors/attendants	89 (59.3%)
Long waiting time (>60-90 min)	76 (50.7%)
Not able to obtain slot for teleconsultation	112 (74.7%)
Trouble in getting OPD facilities due to limited OPD patient intake	80 (53.3%)
Not able to avail chemotherapy in daycare	38 (25.3%)
Not able to avail radiotherapy in daycare	9 (6%)
Deferment of surgery	22 (14.7%)
Visit private hospital for cancer treatment due to time constrain	21 (14%)

Other problems faced by cancer patients

Table 2 represents personal issues encountered by cancer patients during the COVID-19 pandemic. As observed during the interviews, at least half of the sampled population did not practice proper mask use; some were using a handkerchief instead of a mask, and most of their masks were below nose level. All the patients were screened for depression using the Patient Health Questionnaire-2 scale. Out of all patients screened, approximately 3/4th of the patients scored less than three on the PHQ-2 Scale, which indicates they had likely gone through at least a mild depressive episode. Around 50% of the patients have a fear of losing their jobs and financial crises, especially because they already have ill health due to cancer, and COVID-19 can put another burden on them. Approximately half of the sampled population experienced the worry of aggravating the underlying illness if there were an inadvertent delay in treatment.

**Table 2:** Personal issues encountered by cancer patients during COVID-19 pandemic

	Frequency % (n=150)
Difficulty practicing proper mask use	77 (51.3%)
Depression due to COVID-19 pandemic	111 (74%)
Encounter any fear of losing one's work amid the economic recession	84 (56%)
Fear of a deterioration of a pre-existing illness, if there will be a delay in treatment	80 (53.3%)

COVID-19 vaccination coverage and status of COVID-19 disease among cancer patients

Table 3 represents the status of COVID-19 vaccination and disease among cancer patients. Out of the total sampled cancer population, 63.3% of the patients (95 out of 150) had an incidence of influenza-like illness (ILI) in the last year, out of which 20.7% (n=31) of the cancer patients tested positive for COVID-19 in the last year. Of those who were examined positive for COVID-19, 1/4th (n=5) of them were hospitalized for COVID-19 care. Out of the total sampled cancer population, less than 1/4th (n=20) of their family members were examined positive for COVID-19 in the last year.

**Table 3:** Status of COVID-19 vaccination and disease among cancer patients

	Frequency % (n=150)	
Taken only one dose of vaccine	32 (21.3%)	
Taken complete two doses of vaccine	24 (16%)	
Not vaccinated	94 (62.7%)	
Reasons for not vaccinated	Do not know about vaccination	15 (10%)
	Do not want to take	16 (10.7%)
	Motivated for vaccination, but yet to get vaccinated	34 (22.7%)
	Doctor asked to get vaccinated once treatment is over	29 (19.3%)
COVID-19 infection, despite having one dose of vaccination	1 (0.7%)	
Any incidence of influenza-like Illness in the last year	95 (63.3%)	
Tested positive for COVID-19 in the last year	31 (20.7%)	
Admitted to hospital for the treatment of COVID-19	5 (3.3%)	
Family member ever examined positive for COVID-19 in the last year	20 (13.3%)	

Equitable access to a safe and effective vaccine is a game-changing tool and is critical to ending the COVID-19 pandemic. Out of the sampled cancer patients, less than 1/4th (21.3%) of the patients got only one dose of vaccine. Less than 1/4th (16%) of the sampled cancer patients got a complete dose of the COVID-19 vaccine. Out of the total sampled population, 62.7% of cancer patients did not even have a single vaccine dose. Of those who got a complete schedule of the COVID-19 vaccine (two doses), none of them got COVID-19 disease post-vaccination. Of those who were not vaccinated yet, 15% of them did not know about vaccination. 15% of the patients who were not vaccinated yet do not want to get vaccinated. Approximately 2/3rd of the patients were motivated to get vaccinated but had not been vaccinated. Less than 1/3rd of the patients were not vaccinated, as the doctor asked them to get vaccinated once treatment was over.

## Discussion

Expert committees like the American Society of Clinical Oncology (ASCO), the European Society for Medical Oncology (ESMO), and the National Comprehensive Cancer Network all have produced recommendations to address cancer patients' consideration of risk versus the benefit of providing treatment amid a pandemic by prioritizing and categorizing people. Illnesses that are medically volatile or extremely life-threatening and situations where the intervention is likely to have a huge impact on overall life or an improvement in the standard of living are included in treatment prioritization recommendations [7,8]. Clinically accepted, these recommendations sometimes pose ethical dilemmas for healthcare professionals weighing the risk of disease progression, chances of metastasis, poor survival, and psychosocial impact on cancer patients. Most cancers can be treated with treatment, yet patients' lives have been put in jeopardy due to treatment inaccessibility. Treatment delays are caused by a variety of factors, making it impossible to predict when treatment will restart [9].

This study describes the journey of cancer patients seeking specific management and care during the COVID-19 pandemic. Even though the pandemic has affected individuals throughout the world, some patient groups have been disproportionately affected. Care for chronic disorders, such as cancer, was somewhat interrupted during the pandemic's peak due to prioritization and resource restrictions.

In the current study, around 3/4th of the sampled population faced a similar transportation problem, which puts them under an extra financial burden. Many patients in our study faced difficulties in obtaining an OPD consultation, getting a slot for telephonic communication, deferred surgeries, having delayed sessions of radiotherapy and chemotherapy, and receiving palliative care. The pandemic has also disrupted cancer research, including basic, experimental, and clinical cancer research, in addition to cancer care delivery [10].

In India, most of the government hospitals were diverted for COVID-19 management, which hampers cancer care delivery. Further, state-of-the-art cancer care equipment owned by the private sector was not affordable for the poorer sections of society [9]. Similarly, our study reported that around 14% of cancer patients had to visit private facilities due to time constraints and delays in appointments, which overburdened them financially.

Our research explored the fact that approximately 74% of our sampled cancer patients experienced a mild episode of depression. The reason behind this may be fear of acquiring COVID-19 infection, fear of deterioration in disease condition due to delay in the treatment course, financial losses, loss of income, and many more. Similarly, during the COVID-19 pandemic, a study found disturbingly high stress and atypically severe symptoms among cancer patients [11].

Furthermore, because of their underlying disease and the immunocompromised state frequently caused by anticancer therapy, cancer patients are at a higher risk of COVID-19 infection. Despite the fact that the magnitude of this risk is unknown, data suggest that COVID-19 is linked to an increased risk of mortality in cancer patients [12]. Patients with malignancies, particularly lung cancer, had a disproportionally increased risk of severe coronavirus disease 2019 (COVID-19) outcomes, such as higher hospitalization and fatality rates, according to several studies [13]. It is unclear if lung cancer or other pre-existing variables like age, genetic diversity in immunity, smoking history, underlying cardiopulmonary illness, or cancer-directed medication predispose a person to severe SARS-CoV-2 infection symptoms [14].

The coronavirus SARS-CoV-2 (COVID-19) pandemic's unique worldwide incidence is unlike any previous seasonal illness. In our study also, oncology patients have been hit especially hard, since they are a particularly vulnerable population in the present pandemic due to their immunocompromised status, which is the result of both cancer and various anticancer therapies. Around 63.3% of our sampled population experienced influenza-like illness last year. Of these, 20% of the patients tested positive for COVID-19. A total of 1/4th of patients who tested positively required hospitalization and intensive care. Also, 13% of the family members tested positive for COVID-19 in the last year. Thus, the virus's high contagiousness was also connected to the risk of caregiver contamination, resulting in a further reduction in healthcare resources [15].

Receiving a cancer diagnosis and enduring treatment is a difficult experience for most individuals. The Coronavirus disease pandemic of 2019 (COVID-19) and subsequent mitigating attempts have aggravated the situation. High hopes for vaccines to return to normalcy in cancer care were critical standpoints in this crisis. The administration of the COVID-19 vaccine to immunocompromised patients was a bit challenging. Only 16% of our sampled population was fully vaccinated, and around 62.7% of them were unvaccinated. This increases the chance of contracting the disease and suffering serious side effects if infected with COVID-19. The reasons behind not getting vaccinated were lack of knowledge, vaccine hesitancy, getting a jab after completion of treatment, and so on. Similar concerns have been raised by Razai et al. in a study as the wider determinants behind the low vaccine uptake in the population [16].

Fear of infection, concerns about the efficacy of COVID-19 therapy, the negative consequences of various mitigation measures (like social isolation), and economic insecurity have all been linked to higher levels of perceived stress in the current study population. Furthermore, social distancing tactics and barriers to care may intensify patients' anxieties and concerns about receiving cancer treatments and a recurrence of the illness. These findings imply that managing such cancer patients becomes even more complicated with limited resources during a pandemic [11]. One of the limitations of our study is the small sample size, which requires further research in terms of a larger sample, different settings, and qualitative aspects to be generalizable.

## Conclusions

The divergence of resources to handle the COVID-19 load has caused delays and disruptions in cancer treatment. Several constraints, such as the lack of transportation services, limited OPD consultations, long waiting times, and difficulty getting telecommunications slots, have hampered cancer care. Moreover, seeking treatment in the private sector due to time or resource constraints at a government facility has overburdened the patient financially. Quarantine, a necessary protocol, also evoked stress and emotional distress in cancer patients. Prioritization of patients by treatment benefits and the intent was applied in most hospital settings. Telemedicine services were used to maintain effective communication between clinicians and the cancer community. Ensuring a proper supply of medicines, curbing vaccine hesitancy, and sensitizing COVID-appropriate behaviours to the cancer community will help them recover from the impact of a pandemic. Further, to negotiate anxiety and panic among cancer patients, caregivers, and healthcare staff, the balance must be maintained by motivating them. Individualized decision-making and adapting technology solutions will help deliver cancer care amid pandemics. Policy reforms must prioritize cancer care in India to maintain a continuum of care, enhance monitoring, increase access, and facilitate patient compliance with the healthcare delivery system.
